# A Protocol for Venous Thromboembolism Prophylaxis in Elective Spine Surgery: A Retrospective Evaluation of a Single-Center Experience

**DOI:** 10.7759/cureus.85297

**Published:** 2025-06-03

**Authors:** Hani Aljohani, Abdulrahman H Alashkar, Ghada K Alkafarnah, Monirh M Almeshigeh, Abdullh A Alsadoon, Amr Alfakhouri

**Affiliations:** 1 College of Medicine, Qassim University, Buraidah, SAU; 2 Department of Surgery, Dr. Sulaiman Al-Habib Medical Group, Buraidah, SAU; 3 Department of Medicine, Dr. Sulaiman Al-Habib Medical Group, Buraidah, SAU

**Keywords:** cervical spine, prophylaxis, rivaroxaban., spine surgery, venous thromboembolism

## Abstract

Introduction: Venous thromboembolic (VTE) events include deep vein thrombosis (DVT) and pulmonary embolism (PE). In spine surgery, the risk of VTE events should be weighed against the risk of developing epidural hematoma (EDH). This has made the subject of VTE prophylaxis in spine surgery an ongoing matter of debate in the scientific community.

Objective: To present our experience with venous thromboembolism prophylaxis in patients undergoing elective spine surgery and discuss it in the light of the literature.

Methods: This was a retrospective review done on all patients who underwent elective spine surgery at our institution from 2019 to 2024 and were prescribed a specified VTE prophylaxis protocol (inpatient enoxaparin and a seven-day course of rivaroxaban following discharge). Patients who underwent spine surgery for non-elective indications, were known to have a malignancy, had a history of VTE, were already on anticoagulant therapy, and/or were non-ambulatory, were excluded.

Results: A total of 528 patients were included; 284 (53.78%) patients were males, and 354 (67.04%) surgeries were thoraco-lumbar. Of the 174 (32.95%) surgeries performed on the cervical spine, 105 were anterior discectomies and 69 were laminectomies. The mean age (±SD) was 55.06 years (±14.9). The majority of the included patients were overweight/obese; 447 patients (84.65%). Of all the patients, 452 (85.6%) started to ambulate on Day 1, and the mean (±SD) length of hospital stay was comparable for patients undergoing cervical and thoracolumbar spine surgeries, 4.6 (±1.3) and 4.8 (±1.6), respectively. None of the patients experienced VTE events, while three (0.56%) developed EDH.

Conclusion: The protocol presented in this report is safe and effective in preventing VTE in ambulatory patients undergoing elective spine surgery. Rivaroxaban is a possible option for VTE prophylaxis in patients undergoing elective spine surgery, including procedures involving the cervical spine.

## Introduction

Venous thromboembolic (VTE) events include deep vein thrombosis (DVT) and pulmonary embolism (PE). They are common complications in surgical patients due to relative immobility and venous stasis. In spine surgery, the risk of VTE events should be weighed against the risk of bleeding, as the development of epidural hematoma (EDH) could lead to permanent neurological deficits [1].

The need for balancing VTE prevention against the risk of EDH has made the subject of VTE prophylaxis in spine surgery an ongoing matter of debate. In this paper, we present our experience in this area and discuss it in the light of the literature. We shall present our VTE prophylaxis protocol implemented with 528 patients and discuss the use of rivaroxaban in spine surgery.

## Materials and methods

Study design, criteria of eligibility and data collection

This is a retrospective study based on the electronic health records (EHR) of patients who underwent any elective spine surgery at Dr. Sulaiman Al-Habib Qassim Hospital within about a six-year-period (between 2019 and 2024).

Inclusion criteria were (1) being 16 years of age or older, (2) had underwent an elective spine surgery at Dr. Sulaiman Al-Habib Qassim Hospital, (3) had been prescribed the VTE prophylaxis described below, and (4) had been followed for a minimum of six months postoperatively. Patients were excluded if (1) the surgery was not elective (e.g. trauma, or cauda equina presentation), (2) they were not prescribed the VTE prophylaxis described below, (3) they were younger than 16 years of age, (4) they were known to have a malignancy, (5) they had a history of a VTE event (DVT or PE), (6) they were on an anticoagulant therapy for any indication, or (7) they were non-ambulatory (bed-ridden) for any reason.

The monthly surgical lists were reviewed, and all patients who underwent elective spine surgery from 2019 were identified. The EHR of the identified patients were screened and assessed for eligibility. The pertinent data of the eligible patients were documented anonymously in an Excel sheet, then reviewed for any errors. The collected data included patients’ characteristics (age, gender, body mass index, medical co-morbidities), surgical data (the surgical procedure, number of vertebral levels, surgical approach, operative time, perioperative blood transfusion) and postoperative information (length of hospital stay, time to mobilization, development of postoperative VTE and/or epidural hematoma). 

VTE prophylaxis protocol

All the patients included in this study were prescribed VTE prophylactic therapy, which consisted of inpatient and outpatient measures. Preoperatively, patients wore thromboembolic deterrent (TED) stockings, which they wore until the time of mobilization postoperatively. Also, they were prescribed low-molecular-weight heparin (LMWH; 40 mg of enoxaparin once daily) starting 24 hours postoperatively and continued for the duration of their hospital stay. Upon discharge, all patients were prescribed a seven-day course of rivaroxaban (10 mg once daily).

Data analysis

After a final review in a Microsoft Excel file, data were imported into SPSS software version 23 for Windows (IBM Corp., Armonk, NY), where descriptive analyses were undertaken. Categorical variables were summarized using frequencies and percentages. Continuous variables were expressed in means and standard deviations (SD).

## Results

A total of 528 patients who underwent elective spine surgeries were reviewed in this study. A total of 284 (53.78%) patients were males, and 354 (67.04%) of the performed surgeries were on the thoraco-lumbar. spine There were 174 (32.95%) surgeries performed on the cervical spine. Of them, 105 (60.34%) were anterior discectomies and 69 (39.65%) were laminectomies.

Table 1 shows the demographic characteristics and comorbidities of the included patients. The mean age (±SD) of the study population was 55.06 years (±14.9); 55.1 years (±16.2) for males, and 54.9 years (±13.3) for females. Of all patients, 447 (84.65%) were either overweight or obese (i.e., had a BMI > 24.9 kg/m2); 228 (80.28%) of males, and 219 (89.75%) of females. The most frequently reported medical comorbidities were diabetes mellitus and hypertension, afflicting 191 (36.17%) and 189 (35.79%) of all patients, respectively.

**Table 1:** Patients’ characteristics, and co-morbidities. SD, standard deviation; BMI: body mass index in Kg/m2; DM, diabetes mellitus; HTN, hypertension; IHD, ischemic heart disease.

Variable	Males (n=284)	Females (n=244)	Total (n=528)
Mean age in years (±SD)	55.1 (±16.2)	54.9 (±13.3)	55.06 (±14.9)
Mean BMI; kg/m^2 ^(±SD)	28 (±4.3)	31.4 (±6.1)	29.75 (±5.5)
BMI categories; n. (%)			
Underweight (BMI < 18.5)	2 (0.7%)	1 (0.4%)	3 (0.56%)
Normal (BMI 18.5-24.9)	54 (19.01%)	24 (9.83%)	78 (14.77%)
Overweight (BMI 25.0-29.9)	145 (51.06%)	76 (31.14%)	221 (41.85%)
Obese (BMI ≥ 30)	83 (29.22%)	143 (58.6%)	226 (42.8%)
Medical co-morbidities; n (%)			
DM	99 (34.85%)	92 (37.7%)	191 (36.17%)
HTN	92 (32.39%)	97 (39.75%)	189 (35.79%)
IHD	35 (12.32%)	8 (3.27%)	43 (8.14%)

Table 2 lists operative and perioperative factors pertinent to the studied population. The vast majority of surgeries involved one to two vertebral levels; 113 of the cervical (64.94%), and 308 (87%) of the thoraco-lumbar surgeries. Postoperatively, 452 (85.6%) of all patients started to mobilize on Day 1; 151 (86%) of the patients who underwent cervical spine surgery, and 301 (85%) of the patients who underwent thoraco-lumbar surgery. Additionally, the mean (±SD) length of hospital stay was comparable for patients undergoing cervical and thoracolumbar spine surgeries, 4.6 (±1.3) and 4.8 (±1.6), respectively.

**Table 2:** Operative and perioperative variables. SD, standard deviation; LOS, length of hospital stay. *Perioperative transfusion is defined as any transfusion of blood products intraoperatively or during hospital stay postoperatively.

Variable	Cervical surgery (n=174)	Thoraco-lumbar surgery (n=354)
Operated levels		
One	57 (32.75%)	230 (64.97%)
Two	56 (32.18%)	78 (22.03%)
More than two	61 (35.05%)	46 (12.99%)
Mean operative time in minutes (±SD)	112.24 (±39.9)	110.7 (±56.8)
Perioperative transfusion* n. (%)	1 (0.57%)	1 (0.28%)
Postoperative mobilization n. (%)		
On the day of surgery	3 (1.72%)	3 (0.84%)
On day 1 postoperatively	151(86.78%)	301 (85.02%)
On day 2 postoperatively or beyond	20 (11.49%)	50 (14.12%)
Mean LOS in days (±SD)	4.6 (±1.3)	4.8 (±1.6)

None of the included patients developed VTE events postoperatively, while three patients (0.56%) developed EDH. One patient developed it during the first 24 hours following a cervical laminectomy, the second was on the third post-operative day following a lumbar laminectomy with fixation and fusion procedure, and the third was on postoperative Day 10 following lumbar laminectomy with fixation and fusion procedure. The third patient developed the EDH following discharge, when he presented to the emergency department with lower limb weakness on the postoperative Day 10. 

## Discussion

VTE events include DVT and PE. They are common complications in surgical patients due to the relative immobility and venous stasis they experience perioperatively. In spine surgery, the risk of VTE events should be weighed against the risk of bleeding, as the development of EDH could lead to permanent neurological deficits [1]. This has made the subject of VTE prophylaxis in spine surgery an ongoing matter of debate.

The incidence of VTE in patients undergoing spine surgery has been reported to be anywhere between 0.3% and 31% [1]. However, the higher figures are reported in studies addressing patients with high baseline VTE risk [2]. The overall risk is generally low. For example, a 2016 meta-analysis that included 34,597 patients found the postoperative VTE risk to be at 2% [3], with some other large studies showing even lower rates [4]. 

On the other hand, the risk of developing EDH has been reported in the literature to be less than 1%. A systematic review that included 16 studies concluded that the overall risk is 0.2% and ranged between 0 and 0.7%. Notably, the review found no difference in the risk of developing epidural hematoma between the studies that did and did not include anticoagulation for VTE prophylaxis [5]. 

The aforementioned figures are comparable to the results of this study. In our study, none of the patients developed VTE events, and three patients (0.56%) developed epidural hematoma. Of these three, one developed it within the first 24 hours following a cervical laminectomy, i.e., prior to commencing any chemical VTE prophylaxis. In other words, our protocol successfully prevented the occurrence of VTE events without increasing the incidence of developing epidural hematoma when compared to the data available in the literature. 

Options for VTE prophylaxis fall under one of two categories: mechanical and chemical. Briefly, mechanical prophylaxis includes TED stockings and pneumatic compression devices, while chemical options include an arsenal of different anticoagulant medications, each with its own advantages and drawbacks. Our VTE prophylaxis protocol described in this study offers several advantages. Starting preoperatively, we used TED stockings and delay initiating chemical anticoagulation until 24 hours postoperatively.

High-quality studies evaluating the use of mechanical anticoagulation in spine surgery are limited. However, it is a non-invasive method that has been shown to be effective in surgical patients in general [6]. Also, the North American Spine Society (NASS) has recommended its routine use in spine surgery [7]. 

The need for chemical anticoagulation in elective spine surgery is debatable, and if given, the ideal timing and duration are not known. The NASS stated in 2009 that for the most commonly performed elective spine surgeries, chemical VTE prophylaxis “may not be warranted.” They indicated that in these surgeries, VTE risk is very low, and the potential benefits of chemical prophylaxis may be confounded by its risks. Also, they indicated that the available literature does not support an ideal timing or duration for chemical VTE prophylaxis [7]. In this study, and with the utilization of chemical prophylaxis, none of our patients developed VTE events, and the risk of developing epidural hematoma was not increased when compared to the figures available in the literature.

In our protocol, we initiated chemical anticoagulation 24 hours postoperatively, starting with enoxaparin for the duration of hospital stay, which for our patients had a mean (±SD) of 4.6 days (±1.3) and 4.8 days (±1.6), for patients undergoing cervical and thoracolumbar surgeries, respectively. Then, we provided a seven-day course of rivaroxaban, which is a direct oral anticoagulant that has the advantage of single oral dosing without the need for laboratory monitoring. By initiating anticoagulation 24 hours postoperatively, we aimed to gain the benefit of prophylaxis while allowing sufficient hemostasis to be achieved, thus minimizing the risk of bleeding-related complications. This finding has been highlighted in a prior study [8].

In general, we have more knowledge and experience regarding the use of LMWH in surgical patients, and data on the use of rivaroxaban in spine surgery is comparatively limited. In a randomized controlled trial (RCT), Du et al. compared rivaroxaban to parnaparin (a LMWH) and found no significant difference between the two in the rates of VTE or bleeding events [9]. In another RCT, Shafiei et al. compared rivaroxaban to enoxaparin and found them to be equally effective at preventing VTE as well as equally safe. However, they recommended discretion in the use of rivaroxaban with cervical laminectomy [10]. In our cohort of patients, 174 patients underwent cervical spine surgeries, of which 69 (39.65%) were laminectomies. Only one case developed an epidural hematoma, which was on the same day of surgery and prior to initiating any anticoagulant therapy. A sample of studies evaluating the use of rivaroxaban in spine surgery is presented in Table 3.

**Table 3:** Studies addressing the use of rivaroxaban for VTE prophylaxis in patients undergoing spine surgery. *These numbers are postoperative medical events defined by the authors to include VTE, CAD, and AP. NA, not available; VTE: venous thromboembolism; CAD: coronary artery disease; AP: aspiration pneumonia.

Study	Study design	Number of participants	Start and duration of anticoagulation	Surgical procedure	VTE cases	Epidural hematoma cases
Du et al. 2015 [9]	Randomized controlled trial	665 patients: 342 in the rivaroxaban group; 324 in the parnaparin group.	For both groups: starting 6-8 h postoperatively for 14 days.	Lumbar spine surgeries	Rivaroxaban: 6 (1.7%); parnaparin: 10 (3.1%)	Rivaroxaban: 1 (0.3%); parnaparin: 0
Zhang et al. 2018 [11]	Retrospective	480 patients: 240 in the rivaroxaban group; 240 in the apixaban group.	Starting: for rivaroxaban group, 6-8 h postoperatively. For apixaban group, 8 am the morning following surgery. Duration for both: 14 days	Thoracolumbar spine surgeries	Rivaroxaban: 9 (3.75%); apixaban: 12 (5%)	NA
Chunhong et al. 2018 [12]	Retrospective	112 patients: 57 in the rivaroxaban group; 55 in the no chemical prophylaxis group.	Starting: 6-10 h postoperatively for 14 days.	Fixation of thoraco-lumbar fractures	Rivaroxaban: 0; no prophylaxis: 2	Rivaroxaban: 0; no prophylaxis: 0
Shafiei et al. 2022 [10]	Randomized controlled trial	244 patients: 123 in the rivaroxaban group; 121 in the enoxaparin group.	For both groups: Starting 24 h postoperatively for 14 days.	Instrumented spine surgery	Rivaroxaban: 8*; enoxaparin: 5*	Rivaroxaban: 3 (2.9%); enoxaparin: 1 (0.8%)

There are two limitations in the study. First, the study design, being retrospective and based on the patients’ EHR, meant that we had no control over the collected data. Second, there was no comparison group against which the presented protocol could be contrasted. 

## Conclusions

The need, timing and type of VTE prophylaxis following elective spine surgeries are still subjects of debate. The protocol presented in this report (utilizing both mechanical and chemical measures) is safe for VTE prophylaxis in ambulatory patients undergoing elective spine surgery. Rivaroxaban is a safe option for VTE prophylaxis in patients undergoing elective spine surgery. This includes procedures involving the cervical spine. 
